# Polynomial Method for PLL Controller Optimization^†^

**DOI:** 10.3390/s110706575

**Published:** 2011-06-27

**Authors:** Ta-Chung Wang, Sanjay Lall, Tsung-Yu Chiou

**Affiliations:** 1 Institute of Civil Aviation, National Cheng-Kung University, No.1 University Road, Tainan 701, Taiwan; 2 Department of Aeronautics and Astronautics, Stanford University, Stanford, CA 94305, USA; E-Mail: lall@stanford.edu; 3 MediaTek Inc., No.1 Dusing Road. 1, Hsinchu Science Park, Hsinchu 30078, Taiwan; E-Mail: tsung-yu.chiou@mediatek.com

**Keywords:** non-linear systems, phase-locked loop, optimization

## Abstract

The *Phase-Locked Loop* (PLL) is a key component of modern electronic communication and control systems. PLL is designed to extract signals from transmission channels. It plays an important role in systems where it is required to estimate the phase of a received signal, such as carrier tracking from *global positioning system* satellites. In order to robustly provide centimeter-level accuracy, it is crucial for the PLL to estimate the instantaneous phase of an incoming signal which is usually buried in random noise or some type of interference. This paper presents an approach that utilizes the recent development in the semi-definite programming and sum-of-squares field. A Lyapunov function will be searched as the certificate of the pull-in range of the PLL system. Moreover, a polynomial design procedure is proposed to further refine the controller parameters for system response away from the equilibrium point. Several simulation results as well as an experiment result are provided to show the effectiveness of this approach.

## 1. Introduction

For nonlinear control systems, one would often like to know the domain of attraction (DoA) of an equilibrium point. Often, this domain is difficult to both find and represent computationally. The usual mathematical tool used for analyzing of the region of attraction is Lyapunov’s method. This gives us a sufficient condition for local stability, although it is often difficult to find a Lyapunov function that can be used as a certificate for the whole DoA. Several prior approaches have used quadratic functions, for example [1–3]. In particular, the approach of [3] makes use of semidefinite programming to find a quadratic function whose sublevel-set is a good inner approximation to the region of attraction. For system in which the DoA is complicated, an ellipsoid may not provide a good approximation, and the above methods leave a large unexplored region within the DoA.

With recent developments in algebra and sum-of-squares techniques, it is now possible to solve for a Lyapunov function with a more general polynomial form [4,5]. Positive definiteness properties are replaced by sum-of-squares(SOS) constraints which can be efficiently solved using convex optimization. The SOSTOOLS [6] toolbox for MATLAB has been developed as an easy computational tool to solve problems that utilizes the SOS techniques. This approach has also allowed finding a Lyapunov function within some specified semi-algebraic region [7,8]. While Lyapunov approach provides a method to certify a given inner approximation to the domain of attraction, it does not immediately provide a way to find it. Tan [9] later extended this concept by using unions of SOS polynomials to estimate the domain of attraction but Tan’s approach needs to solve a bilinear optimization. The level-set method [10] has been developed to find a semi-algebraic representation of the DoA by semidefinite programming. With these polynomial techniques, it is possible to precisely estimate the DoA of a nonlinear polynomial system and to find a suitable Lyapunov function as the stability certificate.

A phase-locked loop (PLL) system is a nonlinear system with limited domain of attraction. Due to its importance in communication systems, analyzing and designing a PLL system has attracted many attentions in this field [11–17]. The current approach for designing a controller for a PLL system is still largely based on the linear model [17]. Hence, the performance of the resultant system cannot be guaranteed at system states far away from the designed equilibrium point.

In this paper, we utilize the current SOS techniques to analyze the domain-of-attraction of a PLL system. A local Lyapunov function can then be found as the certificate of the DoA using the proposed approach. The Lyapunov function will be further used to improve the stability region and performance of the PLL system. Using this approach, it is possible to design a PLL with a predefined form of controller that has larger domain-of-attraction than the linear design approach. Moreover, since we are designing the system in the nonlinear region, system dynamics outside the linear region can be further refined. Examples of second order PLL systems are used later in this paper to show the effectiveness of this design approach. In the provided examples, we demonstrated that the system designed by the proposed method has 20% larger domain-of-attraction and less overshoot with faster convergent rate than the linear design approach.

This paper is organized as follows. Section 2 contains some preliminary knowledge about SOS techniques. The advection algorithms as well as the SOS methods for finding a local Lyapunov function are stated in Section 3. A brief discussion of the configuration of a PLL can be found in Section 4 and the proposed method for analyzing and designing a PLL controller are presented in Section 5. Then the paper is summarized in Section 6.

## 2. Preliminaries

The following are some definitions that will be used frequently in this paper. ℝ[*x*] is used to represent the ring of polynomials in *x* with real coefficients. A polynomial *f* ∈ ℝ[*x*] is called positive semidefinite (PSD) if *f*(*x*) ≥ 0, for all *x* ∈ ℝ*^n^*. A polynomial *f* is called SOS if there exist polynomials *g*_1_, ..., *g_s_* ∈ ℝ[*x*] such that $f=g_{1}^{2}+g_{2}^{2}+\cdots +g_{s}^{2}$. Clearly if *f* is SOS then *f* is PSD. It is also well-known that the converse is not true. Σ denotes the set of all SOS polynomials in ℝ[*x*]. ℝ_+_ is used to represent the set of nonnegative real numbers. 𝒝*_r_* is used to represent the open ball with radius *r* centered at the origin.

Suppose *g* : ℝ*^n^* → ℝ is *C*^1^. Define the 0*-sub-level set* of *g* to be *sub*(*g*) ⊂ ℝ*^n^* given by *sub*(*g*) = {*x* ∈ ℝ*^n^* | *g*(*x*) ≤ 0}. Further define the boundary of *sub*(*g*) as ∂ *sub*(*g*).

One feature of the proposed advection algorithm is that the advection problem can be converted into a semidefinite program. The following is a standard form of a semidefinite program.

$$\begin{array}{ccc} \underset{X}{\text{min}} & \text{trace}(CX) & \\ \text{s}.\;\text{t}. & \text{trace}(A_{i}X)=b_{i} & \text{for }i=1,\ldots ,m\\ & X≽0, & \end{array}$$

where *X* ∈ ℝ^*n*×*n*^ is symmetric. *X* ≽ 0 means that *z^T^* *X z* is positive semidefinite for all *z* ∈ ℝ*^n^*.

The condition of one semi-algebraic set containing another semi-algebraic set is one of the key constraints used in this paper. The following lemma shows that this kind of relationship can be converted to constraints on the coefficients of the polynomials. The proof can be found in [4] or [7].

**Lemma 1.** *Given p*, *q* ∈ ℝ[*x*], *suppose there exist s*_0_, *s*_1_ ∈ Σ *such that*

(1) $$\begin{array}{ll} s_{0}-s_{1}q+p=0 & \mathrm{for all}\;x\in {\mathbb{R}}^{n} \end{array}\;\;$$

*Then sub*(*q*) ⊂ *sub*(*p*). *Further, given q and the degree bound of p*, *s*_0_, *and s*_1_, *the set of coefficients of p*, *s*_0_ *and s*_1_ *satisfying* (1) *is the feasible set of a semidefinite program*.

*Proof*. See, for example, [4] or [7].

The representation shown in Lemma 1 is one of the simplest cases of Schmüdgen’s Theorem [18]. Schmüdgen’s Theorem states that if *p* ∈ ℝ[*x*] is strictly positive inside a compact semi-algebraic set *S* generated by *p*_1_, . . ., *p_m_* as *S* = {*x* ∈ ℝ*^n^* | *p_i_* ≥ 0, *i* = 1, 2, . . ., *m*}, then

$$p=\Sigma_{v}p_{1}^{v_{1}}\ldots p_{m}^{v_{m}}s_{v}$$

where *v* = (*v*_1_, . . ., *v_m_*) ∈ {0, 1}*^m^* and *s_v_* ∈ Σ. Putinar [19] later showed that under some additional constraints on *p_i_*, *p* has a simpler representation as

$$p=s_{0}+s_{1}p_{1}+\ldots +s_{m}p_{m}$$

The gap between Schmüdgen’s and Putinar’s representation is later investigated by Jacobi and Prestel [20]. In the simple case shown in Lemma 1, if *sub*(*q*) ⊂ *sub*(*p*) and *sub*(*q*) is compact, the representation of *p* by (1) is always possible.

The following result is similar. Given *q* ∈ ℝ[*x*], if there exists *s*_0_, *s*_1_ ∈ Σ and ɛ > 0 such that

$$s_{0}+s_{1}q-p+ɛ=0$$

then *sub*(*p*) ⊂ *sub*(*q*).

Usually *q* is a given polynomial and *p* is the solution to find such that *sub*(*p*) and *sub*(*q*) approximately represent the same set with some other constraints on *p*, such as having lower degree or passing through several pre-specified points. The above results are used to construct such constraints.

## 3. Acquiring the Local Lyapunov Function

Finding a local Lyapunov function is coupled with finding the DoA. Without a clear knowledge of the actual shape of the DoA, it is hard to find a Lyapunov function that can be used to represent the entire DoA [4,8]. To deal with this difficulty, we utilize the current development in set advection [10].

### 3.1. Set Advection

In this paper, we will consider the following autonomous system

(2) $$\dot{x}(t)=f(x)$$

where *f* : ℝ*^n^* → ℝ*^n^* is locally Lipschitz. From the basic local existence and uniqueness theorem [21], given an open subset *U* ∈ ℝ*^n^*, there exist *c* ∈ ℝ_+_ such that the autonomous system (2) has a unique solution for any *z* ∈ *U* in the compact time interval [−*c*, *c*].

We define the *flow map φ_t_* : ℝ*^n^* × ℝ → ℝ*^n^* to be the local unique solution of

$$\begin{array}{l} \frac{\partial \varphi_{t}(z)}{\partial t}=f(\varphi_{t}(z))\;\text{for }t\;\in [-c,c],c(z)\in {\mathbb{R}}_{+},z\in {\mathbb{R}}^{n}\\ \;\;\;\varphi_{0}(z)=z \end{array}$$

For any *t* ∈ ℝ such that *φ_t_*(*x*) exists, the map *φ_t_* : ℝ*^n^* → ℝ*^n^* is continuous, invertible and has a continuous inverse, *i.e.*, it is a topological homeomorphism on ℝ*^n^* [22].

**Figure 1.**
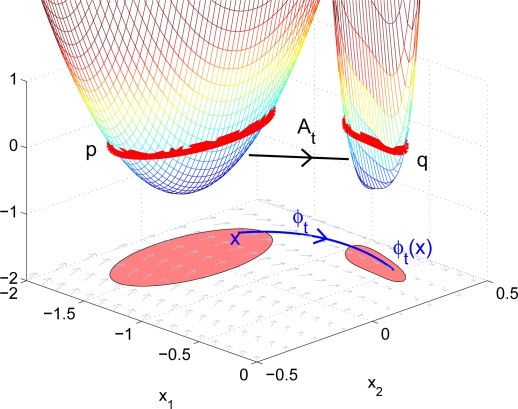
The advection operator *A_t_*.

Given *t* ∈ ℝ, we define the time *t* advection operator *A_t_* : *C*(ℝ*^n^*, ℝ) → *C*(ℝ*^n^*, ℝ) by

$$\begin{array}{lll} q=A_{t}p & \text{if} & q(x)=p(\varphi_{-t}(x))\;\text{for all }x\in {\mathbb{R}}^{n} \end{array}$$

where *C*(*X*, *Y*) is the set of functions mapping from *X* to *Y*. The map *A_t_* is also called the Liouville operator associated with *f*. A very important property is that it is linear. Figure 1 shows the concept of the advection operator. Given polynomial *p*, *A_t_* maps the coefficients of *p* to another polynomial *q* such that *sub*(*q*) = *φ_t_* *sub*(*p*). We relate the advection operator to the advection of sets in the following remark.

**Remark 1.** *Suppose g*_1_, *g*_2_ *are functions mapping* ℝ*^n^* *to* ℝ. *If g*_2_ = *A_t_g*_1_ *then sub*(*g*_2_) = *φ_t_* (*sub*(*g*_1_)).

### 3.2. Time-Stepping

Since we are performing advection, we must use an approximation to the flow map *φ_h_* with time step *h*. Many such approximations are possible, and are provided by the theory of numerical integration. The first-order Taylor approximation to *q* = *A_h_p* is the map *B_h_* : *C*(ℝ*^n^*, ℝ) → *C*(ℝ*^n^*, ℝ) given by

$$\begin{array}{ll} q=B_{h}p & \text{if} \end{array}q(x)=p(x)-hDp(x)f(x)$$

where the derivative *Dp*(*x*) is a linear map *Dp*(*x*) : ℝ*^n^* → ℝ*^n^* at each point *x*.

Based on the required accuracy of the advection, we could also choose to use higher order Taylor approximation. However, depending on the system dynamics, this usually will lead to the requirement of using higher degree polynomials in the sum-of-squares constraints. The relationship between the accuracy and the degree of polynomials will be further investigated in future work.

### 3.3. Domain-of-Attraction Estimation

The set advection concept is used to estimate the DoA of a system. We use the following definition of the DoA in this paper.

**Definition 1.** *Suppose f* : ℝ*^n^* → ℝ*^n^ is analytic with the flow map, φ, and the origin is asymptotically stable. Define the* domain-of-attraction *(also called the basin/region of attraction) of f to be R* ⊂ ℝ*^n^ such that for any x* ∈ *R*, *φ_t_*(*x*) *is defined for all t* ≥ 0 *and* lim_*t*→∞_ *φ_t_*(*x*) = 0.

The following properties can be easily derived. The detailed proofs can be found in [10].

**Lemma 2.** *Suppose f is analytic and the origin is asymptotically stable and R* ≠ ∅. *Suppose also S*_1_ ⊂ *R and* 0 ∈ *S*_1_, *and S*_1_ *is a connected closed positively invariant set. Let h* > 0 *be a positive constant, and define the* backward advection *of S*_1_ *to be S*_2_, *given by*

$$S_{2}=\varphi_{-h}S_{1}$$

*Then S*_1_ ⊂ *S*_2_ ⊂ *R, and S*_2_ *is also connected, closed and positively invariant. Furthermore, ∂S*_2_ = *φ*_−*h*_*∂S*_1_.

**Theorem 1.** *Suppose f is analytic and the origin is asymptotically stable and h* > 0. *Also suppose* 0 ∈ *S*_0_ *and S*_0_ ⊂ *R is a closed connected positively invariant set, such that there exists ε* > 0 *such that* 𝒝*_ε_* ⊂ *S*_0_.

*Define the sequence of sets S*_0_, *S*_1_, *S*_2_, . . . *by*

$$\begin{array}{ll} S_{k+1}=\varphi_{-h}S_{k} & \mathrm{for}\;k=0,1,2,\ldots \end{array}$$

*Then this sequence converges to R in the following sense:*

- *S_k_* ⊂ *R for all k* ∈ ℕ.
- *S_k_* ⊂ *S*_*k*+1_ *for all k* ∈ ℕ.
- *For every x* ∈ *R, there exists n such that x* ∈ *S_n_*

### 3.4. Star-Shaped Constraint

For the case of estimating the DoA, we introduce the concept of star-shaped sets. The star-shaped sets have many important properties and can be easily implemented as a semidefinite program. We now start with the first property. The detailed information about the star-shaped set can be found in [10].

**Definition 2.** *A set S* ∈ ℝ*^n^* *is called* star-shaped *if for all x* ∈ *S*

$$\begin{array}{ll} \lambda x\in S & \mathrm{for all}\;\lambda \in [0,1] \end{array}$$

*The set S is called* strictly star-shaped *if for all x* ∈ *S*

λx∈int(S)  for all λ∈[0,1)

Note that a star shaped set *S* is connected. We now give a simple sufficient condition that ensures a sub-level set is star-shaped. We make the following definition.

**Definition 3.** *Suppose g* : ℝ*^n^* → ℝ. *We call g* strictly star-shaped *if g is C*^1^ *and further satisfies g*(0) < 0 *and*

$$\begin{array}{ll} \mathrm{Dg}(x)x>0 & \mathrm{for all}\;x\neq 0 \end{array}$$

The following lemma shows the connection between strictly star-shaped functions and star-shaped sets.

**Lemma 3.** *Suppose g* : ℝ*^n^* → ℝ *is strictly star-shaped. Then sub*(*g*) *is strictly star-shaped.*

*Proof.* Suppose *x* ∈ *sub*(*g*). Let *y* : ℝ_+_ → ℝ*^n^* be the function

$$y(t)=e^{-t}x$$

The trajectory of *y*(*t*) follows the straight line connecting *x* and the origin. We would like to show that *y*(*t*) ∈ *sub*(*g*) for all *t* ≥ 0. We have

$$\begin{array}{lll} \frac{d}{dt}g(y(t)) & = & -\mathrm{Dg}(y(t)y(t))\\ & < & 0 \end{array}$$

for all *t* ≥ 0. Also

$$g(y(t))-g(y(0))=\int_{0}^{t}\frac{d}{dt}g(y(t))\;dt$$

and since *y*(0) = *x* we have *g* (*y*(*t*)) < 0 for all *t* ≥ 0 as desired.

Now we need to show that if *y* ∈ ∂ *sub*(*g*) then there does not exist *λ* ∈ [0, 1) such that *λy* ∈ ∂ *sub*(*g*). Suppose for the sake of a contradiction that there does exist such a *y* and *λ*. We know that there exists such *λ* > 0, since *g*(0) < 0. Define the function *h* : [0, 1] → ℝ by

$$\begin{array}{ll} h(\theta )=g(\theta y) & \text{for} \end{array}\theta \in [0,1]$$

Then the derivative of *h* is

$$\begin{array}{lll} h^{\prime }(\theta ) & = & \frac{1}{\theta }Dg(\theta y)(\theta y)\\ & > & 0 \end{array}$$

for all *θ* ∈ (0, 1). From the assumptions we know *h*(*λ*) = 0 and *h*(1) = 0. Since *h* is *C*^1^ on [*λ*, 1], by the mean-value theorem there must exist *θ* ∈ (*λ*, 1) such that *h*′(*θ*) = 0, which is a contradiction.

For the purposes of this paper, we would like to construct a convex set of functions whose sub-level sets are connected. Although the convex set of all convex functions on ℝ*^n^* will suffice, using it would unnecessarily restrict the class of sets describable to be convex. One cannot simply use the set of all functions whose 0-sub-level set is connected, since this set of functions is not convex. We therefore choose the set of *strictly star-shaped* functions, which is a convex set. We will use strictly star-shaped polynomials to represent sets. This is significantly more general than existing approaches using quadratic functions [1–3]. Also, it has been shown in Lemma 3 that if *g* is strictly star-shaped, then *sub*(*g*) is strictly star-shaped. By using this property, we can easily pose the star-shaped constraints on *g* to make *sub*(*g*) a connected set.

### 3.5. An Algorithm for Backward Advection

Here we will state the result of the backward advection algorithm. Given a strictly star-shaped polynomial *g*_*i*−1_ such that *sub*(*g*_*i*−1_) ⊂ *R*, and *sub*(*g*_*i*−1_) is bounded and positively invariant, we compute a polynomial *g_i_* such that *sub*(*A_h_g_i_*) ≈ *sub*(*g*_*i*−1_) as follows.

Pick *α* > 0 and positive integer *d*. Solve, using semidefinite programming, the following feasibility problem for *g_i_* ∈ ℝ[*x*], *s*_1_, *s*_2_, *s*_3_, *s*_4_ ∈ Σ.

$$\begin{array}{lll} g_{i}(0) & = & -1\\ Dg_{i}(x)x & > & 0\\ s_{3}-s_{4}g_{i-1}+B_{\left(h-\alpha \right)}g_{i} & = & 0\\ s_{1}+s_{2}g_{i-1}-B_{h}g_{i} & = & 0\\ \text{deg}(g_{i}) & \leq & d \end{array}$$

Here we introduced an important parameter, *α*, which we think of as follows. The above algorithm finds a degree *d* polynomial *g_i_* such that *g_i_* is strictly star shaped, *φ_h_* *sub*(*g_i_*) ⊂ *sub*(*g*_*i*−1_), and *φ_h−α_* *sub*(*g_i_*) ⊂ *sub*(*g*_*i*−1_). Hence the parameter *α* may be thought of as a tolerance that allows for the constraint that *g_i_* is required to have degree *d* or less. Then from the result of Theorem 1, lim_*i*→∞_ *sub*(*g_i_*) converges to the domain-of-attraction. It should be noted that this technique only works in the case that the advected set is positively/negatively invariant.

### 3.6. Stopping Conditions

By using the proposed level-set method, one can successfully propagate the system states backward in time. However, a stopping criterion is still needed to terminate the iterations. To detect the convergence of the advected sets, the closeness of two semi-algebraic sets is analyzed. The following result shows that the closeness of two semi-algebraic sets can be estimated by using scaled sets. The detailed proof can be found in [10].

**Theorem 2.** *Suppose g*_1_ *and g*_2_ *are strictly star-shaped functions, sub*(*g*_1_) ⊂ *sub*(*g*_2_), *and sub*(*g*_2_) *is bounded. Suppose x*_1_, *x*_2_ ∈ ℝ*^n^* *are two points such that*

$$\begin{array}{lll} x_{1}\in \partial \;sub(g_{1}) & and & x_{2}\in \partial \;sub(g_{2}), \end{array}$$

*and x*_1_ = *αx*_2_ *for some α* ≥ 0. *Define the function q* : ℝ*^n^* → ℝ *by*

$$\begin{array}{ll} q(x)=g_{2}(\lambda x) & \mathrm{for all}\;x\in {\mathbb{R}}^{n} \end{array}$$

*where λ* > 1. *Then if sub*(*q*) ⊂ *sub*(*g*_1_),

$$\frac{\left||x_{2}-x_{1}|\right|}{\left||x_{2}|\right|}\leq 1-\lambda^{-1}$$

**Figure 2.**
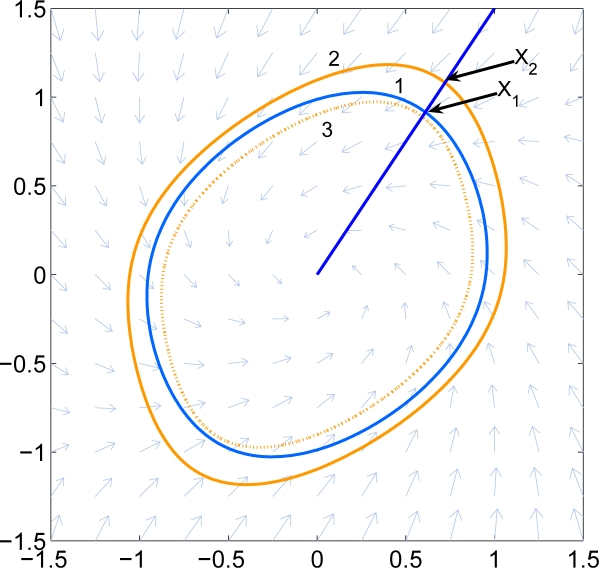
Example of stopping conditions. Curve 1 is the inner set and curve 2 is the outer set. Curve 3 is the shrunk set of the outer set.

To determine when the algorithm should terminate, one formulates an optimization problem using Lemma 1 to determine the smallest *λ* > 1 such that *sub*(*q*) ⊂ *sub*(*g*_2_), where *q*(*x*) = *g*_2_(*λx*). Again, this may be evaluated using semidefinite programming. In practice, one picks a *λ* > 1 in advance, and checks this condition after each iteration. Figure 2 shows an example. Here Curve 1 is *∂ sub*(*g*_1_), Curve 2 is *∂ sub*(*g*_2_), and Curve 3 is ∂ *sub*(*q*). The largest radial deviation between Curves 1 and 2 is less than 0.3.

### 3.7. The Local Lyapunov Function

We find a local Lyapunov function in order to construct an initial star-shaped positively invariant set. The following result is standard.

**Proposition 1.** *Suppose f* : ℝ*^n^* → ℝ*^n^* *is analytic and the origin is a stable equilibrium point. Also suppose V* : ℝ*^n^* → ℝ *is a C*^1^ *function, γ* > 0, *and the set*

$$D_{\gamma }=\{x\in {\mathbb{R}}^{n}|V(x)\leq \gamma \}$$

*is compact. Further suppose*

$$\begin{array}{ll} V(x)>0 & \mathrm{for all}\;x\neq 0\\ V(0)=0 & \\ DV(x)f(x)<0 & \mathrm{for all}\;x\neq 0,x\in D_{\gamma } \end{array}$$

*Let g*_0_(*x*) = *V* (*x*) − *γ. Then sub*(*g*_0_) *is positively invariant, and sub*(*g*_0_) ⊂ *R*.

One simple approach to finding an initial sub-level set is to find a quadratic Lyapunov function for the linear model of the system, and use a small sub-level set of this quadratic polynomial as the initial set.

An alternative method which often gives a much larger initial set is as follows. Choose a polynomial *p* ∈ ℝ[*x*] such that *sub*(*p*) ⊂ *R*. We then solve the following convex feasibility problem. Find *V* ∈ ℝ[*x*] and *s*_0_, *s*_1_ ∈ Σ such that

$$\begin{array}{ll} DV(x)x>0 & \text{for all}\;x\neq 0\\ V(x)>0 & \text{for all}\;x\neq 0\\ V(0)=0 & \\ DV(x)f(x)+s_{0}-s_{1}p=0 & \text{for all}\;x\neq 0 \end{array}$$

Similar methods for finding local Lyapunov functions along with details on the construction of the associated semidefinite program may be found in [5,8]. Here we have added the first constraint to ensure that *V* − *γ* is strictly star-shaped for *γ* > 0. Note that these constraints imply that all sub-level sets of *V* are compact. Given *V*, we then solve the convex program

$$\begin{array}{lll} \text{maximize} & \gamma & \\ \text{subject to} & V-\gamma -s_{0}-s_{1}p-ɛ=0 & \text{for all }x\\ & s_{0},s_{1}\in \Sigma & \end{array}$$

where *ε* > 0 is small. The optimal *γ* satisfies *sub*(*V* − *γ*) ⊂ *sub*(*p*). Then *V* and *γ* satisfy the assumptions of Proposition 1 and so we may use *g*_0_ = *V* − *γ* as the function defining our initial level-set.

After we have found an estimate of the DoA, we can then use Proposition 1 to find a Lyapunov function that can be used to describe the behavior of the system within the DoA. To adequately describe the system behavior, this approach requires that we have a good estimate of the DoA. The following examples show that the level-set algorithm can precisely estimate the DoA.

### 3.8. Examples of DoA Estimation

**Example 1.** *Consider the following dynamical system*

$$\begin{array}{l} \dot{x}=0.5y-x(1-x^{2}-0.25y^{2})\\ \dot{y}=-x-0.5y(1-x^{2}-0.25y^{2}) \end{array}$$

**Figure 3.**
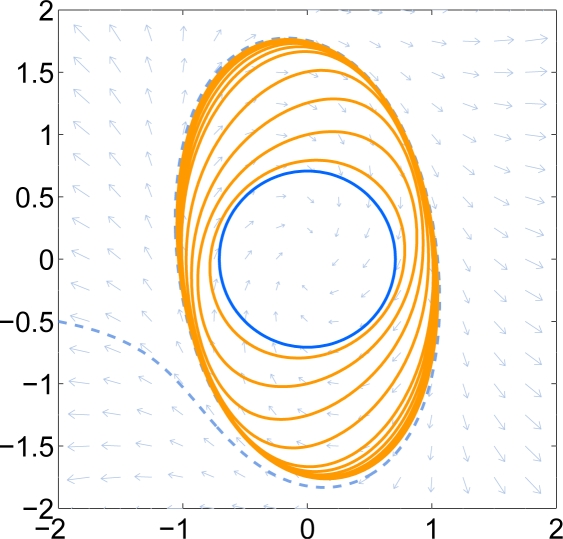
Successive iterates of the level-set algorithm.

*The origin is a locally stable equilibrium point. Here we start with the initial polynomial g*_0_ = 2*x*^2^ + 2*y*^2^ − 1. *The results of the level-set method are shown in Figure 3, using time step h* = 0.2. *It can be seen that the successive iterates approach the true DoA.*

**Example 2.** *Consider the Van der Pol oscillator with inverted time:*

$$\begin{array}{l} \dot{x}=-y\\ \dot{y}=x-y(1-x^{2}) \end{array}$$

**Figure 4.**
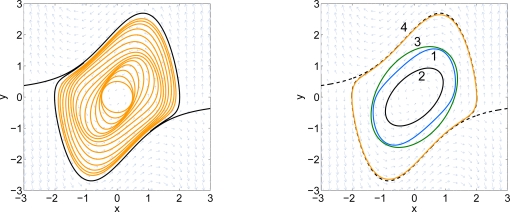
Van der Pol oscillator. The left figure shows the sequence of iterations. The right figure shows the final iterate as Curve 4 along with some other results. Curve 1 is ∂ *sub*(*g*_0_), when *g*_0_ is obtained through the semidefinite programming based procedure in Section 3.7. For comparison, Curve 2 is the result of [2] and Curve 3 is the result of [1].

*This system is locally stable around the origin. An initial sub-level set given by the quadratic polynomial g*_0_ = 4*x*^2^ + 4*y*^2^ − 1 *is used which can be verified to be positively invariant. A time step of h* = 0.2 *is used. The time tolerance parameter α is* 0.02*. The even-numbered iterates g*_0_, *g*_2_, *g*_4_, . . . *are shown in Figure 4. For reference, some of the iterates are listed below and normalized to allow integer coefficients.*

$$\begin{array}{lll} p_{2} & = & -1,000+2,252y^{2}-88y^{4}+11y^{6}-907xy-56xy^{3}-4xy^{5}+3,883x^{2}\\ & & +360x^{2}y^{2}-57x^{2}y^{2}+660x^{3}y-x^{3}y^{3}-417x^{4}+21x^{4}y^{2}+81x^{5}y+260x^{6}\\ p_{4} & = & -1,000+1,614y^{2}-137y^{4}+16y^{6}-1,654xy-170xy^{3}+14xy^{5}+3,162x^{2}\\ & & +480x^{2}y^{2}-43x^{2}y^{4}+94x^{3}y-35x^{3}y^{3}+144x^{4}-2x^{4}y^{2}+192x^{5}y+335x^{6}\\ p_{28} & = & -10,000+2510y^{2}-56y^{4}+2y^{6}-4,306xy+42xy^{3}+4xy^{5}+4,099x^{2}\\ & & +25x^{2}y^{2}+2x^{2}y^{4}+1,103x^{3}y-27x^{3}y^{3}-687x^{4}-x^{4}y^{2}+2x^{5}y+84x^{6} \end{array}$$

*It can be seen that the iterates gradually approach the exact boundary of the DoA. After* 30 *iterations, the solution covers most of the stable region.*

*After* 40 *iterations, the stopping criteria allowing an absolute radial change of* 0.01 *has been met. The final result is shown in Figure 4 as Curve* 4. *Curve* 1 *is ∂*𝒞(*g*_0_) *if we were using the semidefinite-programming based procedure in Section 3.7. For comparison, Curve* 2 *is the result of [2] and Curve* 3 *is the result of [1]*.

**Example 3.** *The following dynamic system is the Example S4 taken from [3].*

$$\begin{array}{l} \dot{x}=-2x+y+x^{3}+y^{5}\\ \dot{y}=-x-y+x^{2}y^{3} \end{array}$$

**Figure 5.**
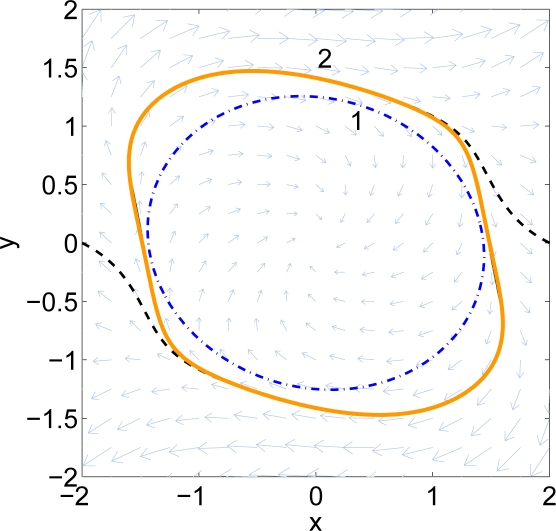
Sub-level sets of *g*_0_ and *g*_20_. The dashed-dot curve is the result from [3] and the solid curve is the result of the advection algorithm.

*Here the initial set is a small circle around the origin. After 20 iterations, the estimated boundary of the DoA has reached the pre-specified bound. The final iterate is shown in Figure 5. The dashed curves show the two system trajectories which are used to represent the true boundary of the domain of attraction. Curve 2 represents the result of the final iterate. Curve 1 is the result from [3].*

## 4. Configuration of a PLL

**Figure 6.**
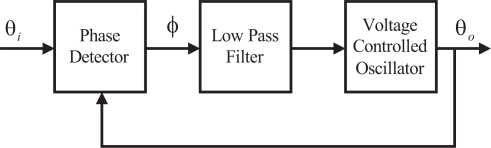
Basic Configuration of a PLL.

Figure 6 shows the basic configuration of a PLL. It has three components; a phase detector, a loop filter, and a *voltage controlled oscillator*(VCO). The VCO generates an output signal whose phase, *θ*_0_(*t*), depends on the phase, *θ_i_*(*t*), of the input signal. The PLL is phase locked when the phase error *φ*(*t*) = *θ_i_*(*t*) − *θ*_0_(*t*) is a constant value and the loop is in stable equilibrium state. Usually, it is desired that the phase error, *φ*(*t*), is maintained at zero.

Of interest is the behavior of the phase error *φ*(*t*). Because of its sinusoidal nonlinearity in the PLL, the phenomenon of chaos is believed to exist [11,12] and its inherent chaotic behavior for broadening the pull-in range of PLL has also been realized [13,14]. A nonlinear controller can drive PLL from chaotic state into periodic state or vice versa [15]. For higher-order PLL, it is not possible to determine whether the loop will or will not slip cycles using the initial frequency alone. In this case, one might define the pull-in range as the separatrix ordinate at *φ* = 0 [17]. Analyzing the DoA of the PLL system provides a better description of the region in which a PLL locks up without slipping. The Lyapunov method has been used for stability analysis in control systems. Here the advection algorithm will be used to find the guaranteed stability boundary of the PLL system and the associated local Lyapunov function is then used to further refine the controller parameters. In [16], a Lyapunov styled analysis for PLL system up to third order is presented. The method shown in this section provides a way to analyze the DoA for a more general system. Also, the form of the Lyapunov function used here is much more flexible.

**Figure 7.**
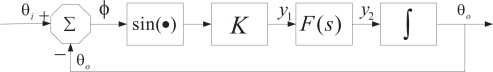
Model of the Phase-Locked Loop.

Figure 7 shows the nonlinear model of the PLL. The sine function here represents the phase detector of the system. *K* in Figure 7 stands for the loop gain of the system. *F* (*s*) is equivalent to the low pass filter shown in Figure 6 and it corresponds to the controller of the PLL. Finally, the integrator in Figure 7 is the voltage or numerically controlled oscillator. The key idea of a PLL system is to use the command, *y*_2_, from *F* (*s*) to steer the oscillator such that *θ*_0_(*t*) tracks *θ_i_*(*t*) as closely and quickly as possible.

### 4.1. Second Order PLL

To use the nonlinear design approach, start with a reference design. The reference design used in this paper is the linear model of a PLL system. A Proportional-Integrator (PI) controller is chosen to be the filter, *F* (*s*), as

(3) $$F(s)=\frac{1+\tau_{2}s}{\tau_{1}s}$$

Using the model shown in Figure 7, it is routine to check that the resulting dynamic equation of the system is

(4) $$\frac{d^{2}\varphi }{dt^{2}}+K\frac{\tau_{2}}{\tau_{1}}\text{cos}(\varphi )\frac{d\varphi }{dt}+\frac{1}{\tau_{1}}K\;\text{sin }(\varphi )=\frac{d^{2}\theta_{i}}{dt^{2}}$$

Assume that the received signal frequency is varying linearly with time and has zero radial acceleration and let *x*_1_ = *φ, x*_2_ = *φ̇*. The PLL system can be rewritten as the following state space model

(5) $$\begin{array}{lll} {\dot{x}}_{1} & = & x_{2}\\ {\dot{x}}_{2} & = & -K\frac{\tau_{2}}{\tau_{1}}\text{cos}(x_{1})x_{2}-\frac{1}{\tau_{1}}\text{sin}\\ & = & k_{1}\;\text{cos}(x_{1})x_{2}+k_{2}\text{sin}(x_{1}) \end{array}(x_{1})$$

Equation (5) is the final nonlinear model of the second order PLL. A linearized model can then be derived as

$$\begin{array}{l} {\dot{x}}_{1}=x_{2}\\ {\dot{x}}_{2}=k_{1}x_{2}+k_{2}x_{1} \end{array}$$

The filter *F* can then be designed using existing linear design approach [17]. One typical choice is to let *ω_n_* = 15, *ζ* = 0.707, *K* = 1, where *ω_n_* is the natural frequency, *ζ* is the damping ratio, and *K* is the overall gain. The two coefficients of the filter are then given as $\tau_{1}=\frac{K}{\omega_{n}^{2}},\tau_{2}=\frac{2\zeta }{\omega_{n}}$

## 5. Phase-Locked Loop Analysis and Design

In this section, the second order PLL controller will be used to demonstrate the nonlinear design approach. The same approach can also be applied to the third order controller design.

### 5.1. Pull-In Range of the Traditional PLL System

**Figure 8.**
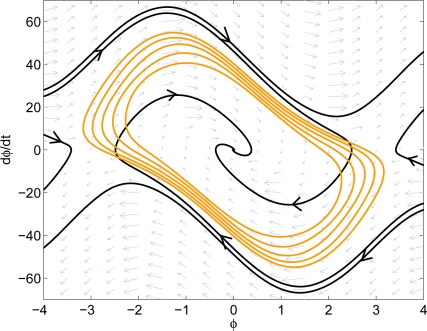
Result of the original PLL system.

Now the advection algorithm can be applied to the PLL nonlinear system. To reduce the required number of iterations, a local Lyapunov function is used as the initial set. After a few iterations of the algorithm, it gives us the estimated domain-of-attraction of the system. The result is shown in Figure 8. Note that the estimated region is based on the Taylor series expansion of the sine and cosine functions. From *Ston-Weierstrass* Theorem, we can approximate the sine and cosine functions to the desired accuracy within a bounded interval. In this example, two degree-10 polynomials are used to approximate sine and cosine functions and the estimated region is only valid between −*π* and *π*. More terms of the Taylor series could also be used to improve the accuracy.

### 5.2. PLL System Controller Design

**Figure 9.**
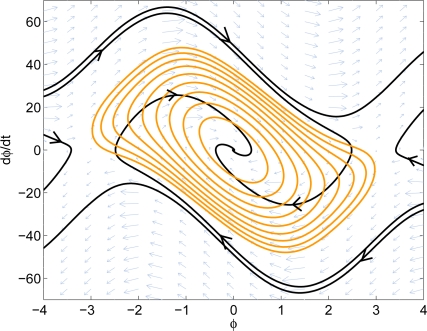
Local Lyapunov level sets of the original PLL system.

After getting a good estimated DoA, a local Lyapunov function that describes the system behavior can be easily computed using Proposition 1. This local Lyapunov function can also be used to describe the trajectories of the system in the DoA. Figure 9 shows several Lyapunov level-sets obtained from the SOS approach.

The SOS techniques can be applied to the design of a controller. For the PLL system, it is desired to design a system which has a larger DoA or faster converging speed. Here, the objective is to find possible system parameters such that the same Lyapunov function is still valid and the system converges faster. This can be done by solving a semidefinite program.

Suppose *V* is a local Lyapunov function for the PLL system. Using the SOS technique, solve the following optimization problem:

(6) $$\begin{array}{l} \text{max }\alpha \\ \;\;\;\text{s}.\text{t}-(DV)f=s_{1}+s_{2}(a-V)\\ \;\;\;\;\;\;\;\;\;\;\;\;\;\;-(DV)f-\alpha q=s_{3}+s_{4}(b-V)\\ \;\;\;\;\;\;\;\;\;\;\;\;\;p(k)\leq 0 \end{array}$$

where *a*, *b* ∈ ℝ_+_ specify the domain of the constraints and *q* is a positive definite performance polynomial specified by the user. *p*(*k*) is a linear constraint of controller parameter *k*. As before, *s*_1_, *s*_2_, *s*_3_, *s*_4_ are SOS polynomials.

Since *V* is now a given function, the above constraints are linear in the controller parameters. The first constraint shows that *V* is a valid Lyapunov function in *sub*(*V* − *a*). This constraint is used to specify the desired DoA to maintain. The second constraint along with the objective function will put an upper bound on the derivative of the Lyapunov function in *sub*(*V* − *b*). Faster decreasing rate implies faster converging speed. This specifies the performance requirement of our system. The user could also put different performance requirements in different sub-level sets of *V*.

Besides dynamic performance constraints, noise bandwidth constraints will be applied as well. The noise bandwidth for this PLL system with PI controller has the following form [17]

$$B_{L}=\frac{\omega_{n}}{2}\left(\zeta +\frac{1}{4\zeta }\right)$$

Assume *ζ* ≥ 1, *ω_n_* ≥ 1. Then

$$B_{L}=\frac{\zeta \omega_{n}}{2}+\frac{\omega_{n}}{8\zeta }\leq -\frac{k_{1}}{4}-\frac{k_{2}}{8}$$

This linear upper bound will be used to find a set of controller parameters that have better dynamic performance while maintaining the same noise bandwidth.

**Figure 10.**
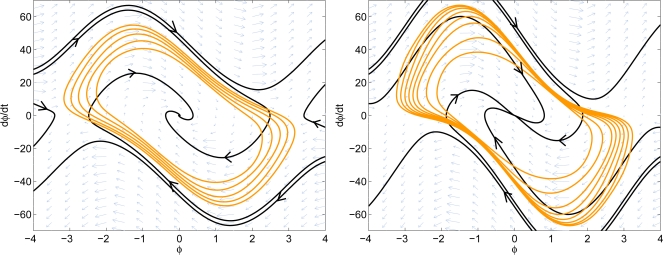
Comparison of the domain-of-attraction. **Left:** linear design. **Right:** nonlinear design.

The system phase portrait as well as the estimated stable region are shown in Figure 10. The nonlinear design has *K* = 1, *ω_n_* = 10.813, and *ζ* = 1.3303. The noise bandwidth is 8.1082 Hz, which is slightly higher than the noise bandwidth of the linear design, 7.9546 Hz. From the phase portrait, the nonlinear design has approximately 20% larger guaranteed domain-of-attraction. It can also be observed from the phase portrait that the nonlinear design has less overshoot than the linear design. This shows that the proposed method increases the system performance while not sacrificing too much of the noise rejection capability.

**Figure 11.**
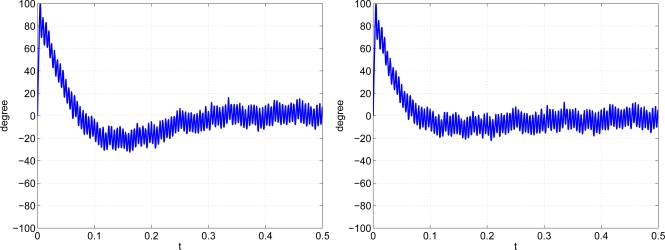
Phase error of Simulink simulation. **Left:** linear design. **Right:** nonlinear design.

A Simulink model is used to compare the nonlinear designed PLL controller with the linear design. In this Simulink model, the sinusoidal input is collapsed by measurement noise and clock noise with zero mean and variances 0.1 and 0.0001, respectively. Figure 11 is the phase error of the two designs. It is clear that the nonlinear designed controller has a much faster convergence rate than the original design.

**Figure 12.**
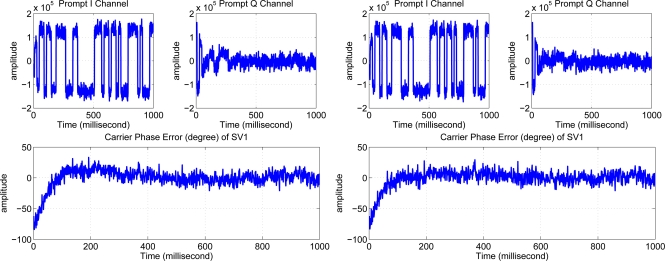
Real GPS experimental results. **Left:** linear design. **Right:** nonlinear design.

Both systems are tested on a NORDNAV R25 software GPS receiver. This receiver collects, down-converts and samples the GPS data by the front end, so that the collected GPS data can be post-processed repeatedly using different tracking-loop filter orders. The results are shown in Figure 12.

It can be seen that the original system has some overshoot and converges around 300 ms. The nonlinear design has much less overshoot and converges about five times faster than the original design.

## 6. Summary

In this paper, we presented a method of designing a PI controller of a PLL system. This design approach is based on the polynomial nonlinear model of the PLL system. This approach starts with the linear design of the controller and then estimates the DoA of the linear designed system to get the suitable local Lyapunov function for the system. The Lyapunov function is then used as the performance constraints to further refine the performance of the system outside the linear region. The DoA of the initial design can also be extended to get a better pull-in region of the PLL system. This approach gives us a way to design a fixed form controller for a nonlinear system.
